# The Fight Against Frostbite Progresses

**DOI:** 10.1021/acscentsci.4c01803

**Published:** 2024-11-06

**Authors:** Ute Eberle

In January 2017, physics professor Hart Bezner was driving home to Waterloo, Ontario, from the remote Arctic hamlet of Tuktoyaktuk when he turned onto the lonely, 724 km Dempster Highway. Outside temperatures hovered around −40 °C. But Bezner, who was wearing gloves, a hat, and an electric jacket plugged into the car’s 12 V outlet, remembers feeling “supremely comfortable.” He was listening to satellite radio and admiring the starlit sky.

Suddenly, Bezner noticed that the car’s heater was blowing cold air, though a gauge showed that the engine’s temperature was climbing.

He stopped, opened the hood, and loosened the cap of the radiator—the system that regulates engine temperature. He recalls a “geyser” of steam and liquid knocking the cap out of his hand and into the darkness. Below it, however, the radiator seemed to have frozen. This prevented coolant from circulating, which caused the engine to overheat.

Bezner slowly drove on. After less than a kilometer the engine had become so hot that he needed to stop, turn off the ignition, and wait for it to cool. Switching between driving and cooling, he crept through the night. His right-hand glove had been soaked with radiator liquid, so he mostly kept it off. With the heater not working, the windshield iced over from the inside, and Bezner says he navigated through “two tiny peepholes” that the defroster kept clear.

It took him 12 h to reach the next city, where he pulled into a motel. So cold that Bezner says he “staggered up the stairs like a drunk, bashing into the railing several times,” he made his way to a room and hot bath. Then the fingers of his right-hand started swelling. By the time Bezner had repaired the car and driven to the city of Whitehorse the next day, those fingers sported big blisters—some of them purple. When the staff at the Whitehorse General Hospital started fussing over him, Bezner realized that he might lose parts of his hand. That is when Bezner first heard of iloprost.

Few people outside the military, the ranks of mountain climbers, and other cold-weather niche circles worry much about frostbite. Yet the number of those affected, though small overall, has been climbing in recent years, partly fueled by extreme sports and outdoor adventuring.

This year brought major progress in the fight against the condition. In February, the US Food and Drug Administration approved the country’s first official treatment for severe frostbite. The drug, iloprost, was originally approved in the US in 2004 for pulmonary arterial hypertension, a type of high blood pressure that affects the lungs. But it has long been shown in other countries to be effective against frostbite. This approval makes the US one of a group of countries where doctors now have a smattering of remedies for the havoc frostbite wreaks on the body.

Some researchers want to go even further than iloprost, though. Rather than treat frostbite after it occurs, they want to provide new chemical tools to prevent the dangerous condition. They are working on “coldscreen” lotions, which they hope will protect skin against freezing similar to how sunscreen protects against ultraviolet rays.

## A chilling condition

When exposed to extreme cold, the body preserves heat by shunting blood to the core. This slows blood flow in the feet and hands from 250 mL/min to as little as 20 mL. Ice crystals form in unprotected skin. With water freezing out, electrolytes become concentrated in the intercellular space, causing surrounding cells to release fluids to rebalance things. The spiky ice crystals puncture cell membranes, and ever more cells become damaged or dehydrated, or rupture.

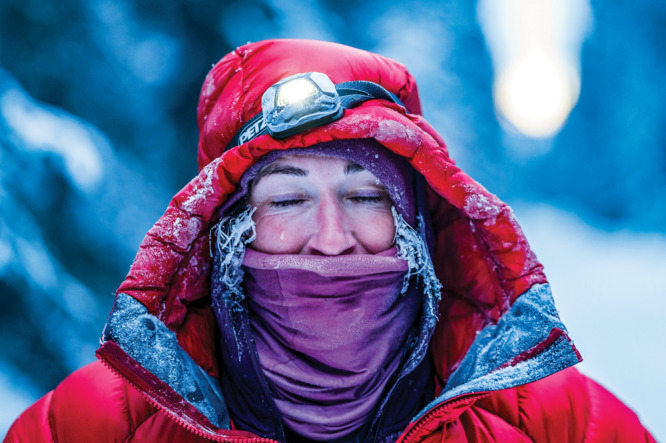

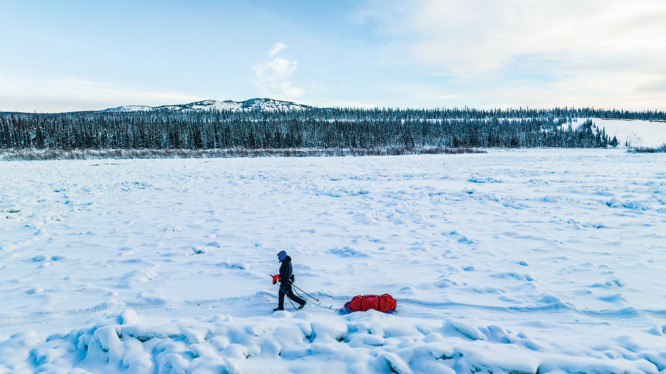





**Figure:**
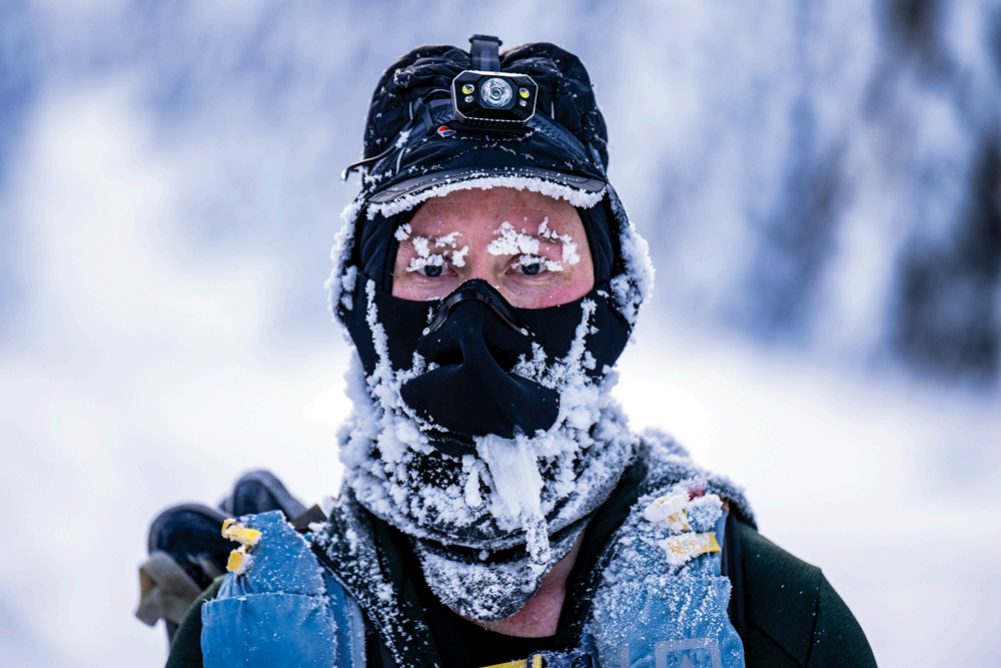
The Montane Yukon Arctic Ultra is a multiday race in which athletes run, bike, or ski long distances through a bitterly cold environment. Alexander Poole gained special permission to use iloprost for frostbite in 2015, before that year’s race. Photos are from the 2023 race. Credit: Mark Kelly

When frostbitten limbs warm up again, blood rushes back into the tissue. With that blood come compounds such as histamines that, triggered by the cell damage, mediate inflammation. The tissue swells, which can compress blood vessels. Small clots often form; as they cut off the oxygen supply, tissue dies and turns black.

The severity of frostbite is often apparent only after days or even weeks. An old medical adage goes “Freeze in January, amputate in July.” Studies show that about a fifth of patients with more severe frostbite lose at least some tissue—and sometimes entire feet or hands.

For centuries, doctors could do very little. Allied forces during World War II recorded 91,000 cases of frostbite. Around that time, it became the practice to rewarm frostbitten limbs in heated water while giving the patient painkillers, such as opioids, against the intense pain this action caused.

That has remained the standard of care until very recently. Some hospitals might also put patients with severe frostbite into a hyperbaric oxygen tank, hoping that the pressure and extra oxygen will reduce the swelling and help the tissue heal. Additionally, doctors have resorted to an off-label use of a stroke medication called tissue plasminogen activator (tPA). A so-called thrombolytic, tPA sticks to the fibrin—the “stringy” parts of the blood that tangle into clots—and breaks it down, thus dissolving such clumps. But to be effective, tPA needs to be given within a few hours of rewarming, and it carries the risk of major complications and death. So despite decades of frostbite injuries, until the advent of iloprost, options remained limited.

## Enter iloprost

Iloprost is a lab-made version of a natural compound in our bodies called prostacyclin. Prostacyclin breaks down quickly—it has a half-life of 42 s—thanks to an enol ether in its structure that hydrolyzes, according to Nicholas Meanwell, a medicinal chemist at the Baruch S. Blumberg Institute. To create a more stable mimic, organic chemists replaced the oxygen atom in prostacyclin’s enol ether with a carbon. But Meanwell says that change caused a significant loss of potency, which was reversed by adding an alkyne and a methyl substituent to one of the new compound’s side chains.

**Figure:**
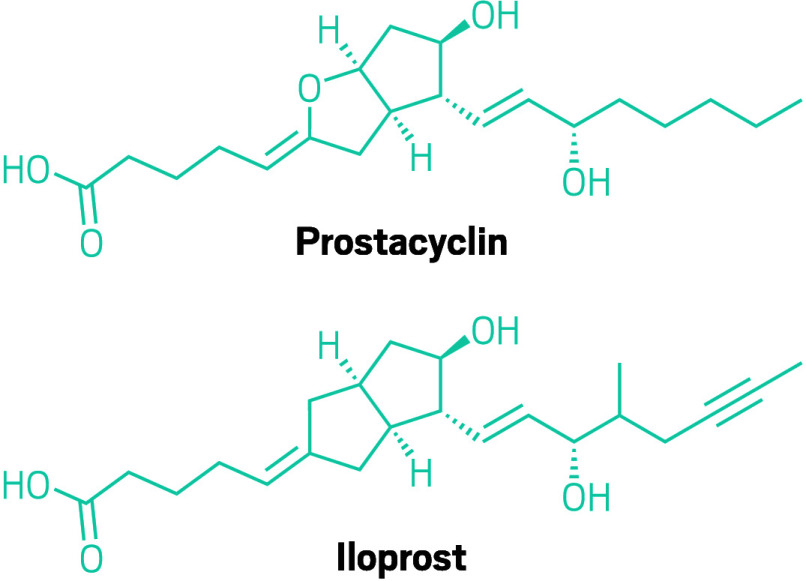
To create iloprost chemists started with prostacyclin—a hormone that occurs naturally in the body—and replaced the oxygen atom in its enol ether with a carbon. They also added an alkyne and a methyl substituent to one of the new compound’s side chains.

When iloprost nestles into prostacyclin receptors expressed in blood vessel walls and platelets, it sets off a chain of reactions that cause blood vessels—particularly small, peripheral ones—to relax and expand while keeping platelets from clotting. Meanwell says that the specifics of iloprost’s binding action are opaque, however, since scientists have so far not decoded the 3D structure of the receptors.

People with pulmonary arterial hypertension inhale iloprost, which widens the blood vessels in the lungs, thus reducing the pressure. For severe frostbite, iloprost is infused intravenously to relax the blood vessels in the extremities. Damaged tissue has a better chance of healing with more oxygen flowing to it.

The drug, whose most common side effects are headaches and facial flushing, was initially used to treat Raynaud’s disease, which makes blood vessels in the fingers and toes constrict. In 1994, a doctor in Austria tried it on five frostbite patients, and none had to undergo amputations. In 2011, a French physician published a small randomized controlled trial in which 16 frostbite patients treated with iloprost had zero amputations, but 9 of 15 patients who did not receive the drug did need amputations. After that, many European doctors started to routinely use iloprost for frostbite. But it would be more than a decade before it was approved in the US as a frostbite treatment. It still has not been approved in Canada.

Bezner didn’t know any of this when he took his blistered hand to the hospital. Nor could he have guessed that, by a lucky coincidence, he would land in the care of one of the first doctors in North America to have started administering iloprost off-label to treat frostbite. Whitehorse General Hospital, which is in the capital of Canada’s Yukon Territory, normally gets about a dozen frostbite patients every winter. (Besides skiers, climbers, and other outdoor enthusiasts, unhoused people are at high risk.) Alexander Poole, a surgeon at Whitehorse General, was irked at how little he could do for these patients. “It was frustrating to do nothing but wait,” he says.

Having heard about iloprost, Poole started pushing to acquire it through Health Canada’s Special Access Program for drugs that haven’t yet been officially approved, since that approval process often requires years. “It can take a while to disseminate medical knowledge, especially in an infrequent condition such as frostbite,” he says.

Poole got permission just before the 2015 Montane Yukon Arctic Ultra, an annual event in which athletes run, bike, or ski for about 692 km. (It will be about 640 km in 2025.) The weather was harsh, with temperatures dipping into the −40s. “People don’t realize how every drop increases their risk,” he says. “If you are prepared for −20, it’s not the same as −40.” Predictably, several athletes suffered frostbite, including a few with fairly serious cases. Poole treated them with iloprost, and they were spared amputations.

**Figure:**
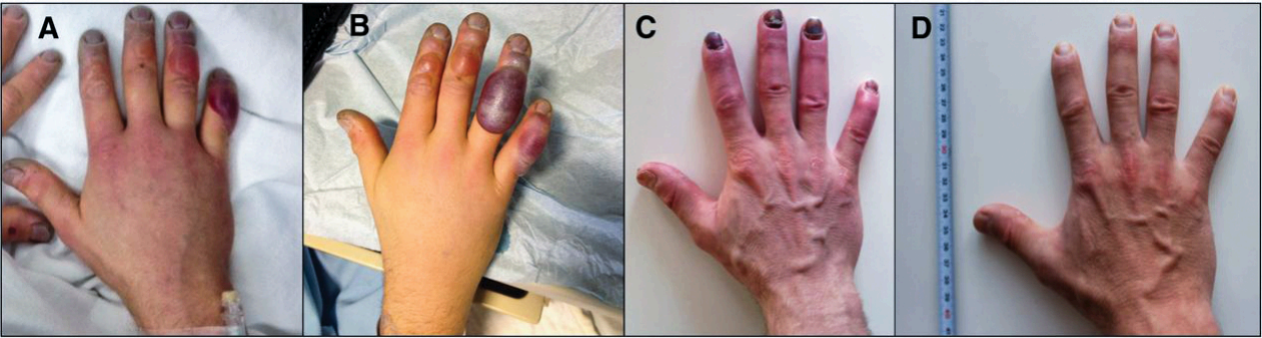
A Montane Yukon Arctic Ultra runner's hand after he was evacuated from the race. Surgeon Alexander Poole and colleagues treated him with iloprost for 5 days. Photos A and B show the hand on day 1 and day 2, respectively, of his hospital stay. Photo C is 1 month after treatment, and Photo D is 6 months after treatment. Credit: *Can. Med. Assoc. J.*, DOI: 10.1503/cmaj.151252

Bezner was put on a 6 h iloprost IV drip for 5 days. The blisters on his hand flattened; the discoloration faded. A month later, he reported in a Canadian Frostbite Care Network blog post that he could use his hand “almost normally” again.

Allison Widlitz, vice president of medical affairs at Eicos Sciences, the California-based company licensed to market iloprost, estimates that fewer than 1,000 people per year in the US will be treated with the drug. Widlitz says that Eicos plans to have iloprost, which it will market under the brand name Aurlumyn, commercially available for the upcoming winter season, but that the cost of the treatment hasn’t yet been determined.

## Staving off frostbite

Munia Ganguli, a biochemist at the Institute of Genomics and Integrative Biology, specializes in methods for delivering genes to the skin and other organs. At a conference, a colleague asked her if these methods could be used as a tactic against frostbite.

Ganguli was intrigued. She knew that animals such as Arctic cod and wood frogs naturally express “antifreeze proteins” in their tissue that allow them to survive subfreezing temperatures. The proteins bind to the lattice of water molecules, which stops ice crystals from forming, Ganguli explains in an email.

In 2015, researchers at Yale University published an experiment in which they transferred genes from a cold-resistant tick to mice. The rodents then expressed the same antifreeze protein in their skin and stayed frostbite-free when the researchers exposed them to freezing water.

Ganguli started more simply. She and her team screened a series of cryopreservative chemicals—synthetic compounds that can protect frozen tissues from damage and are used to preserve, for example, sperm and embryos in fertility clinics. For the purpose of preventing frostbite, she settled on two: dimethyl sulfoxide, which binds to water molecules, stopping them from forming lattices in cells; and poly(vinyl alcohol), which does something similar in the extracellular space. Both substances are routinely used to preserve red blood cells in storage, Ganguli says.

Her team mixed the compounds with an aloe vera base, applied the cream to the skin of mice, left it on for 15 min, then touched the animals’ backs with icy-chilled magnets. Ganguli was surprised how well the cream protected the mice from frostbite. “We used some very simple off-the-shelf chemicals—I think it was lucky that we hit the right combination.”

More work is needed. So far, the protective effect seems to last only about 15 min. Ganguli also needs to run tests on human subjects. She wants to eventually try antifreeze proteins instead of cryopreservative chemicals. “There is so much to explore,” she says. “We are at the tip of the literal iceberg.”

Bezner, who long ago fell in love with Tuktoyaktuk and has visited it many times, says he finds the idea of coldscreens “interesting” but would need to know more before deciding whether to use them. Still, those products are most likely years away. Ultimately, Bezner was undeterred by his brush with frostbite, although reminders of the damage lingered for some time. His fingernails stopped growing for months. And Bezner says that for years, whenever he reached into his freezer he felt “almost instant pain.” Studies show that about two-thirds of frostbite patients suffer long-term effects, including neuropathy, chronic pain, cold hypersensitivity, numbness, and arthritis. There are indications that iloprost might prevent some of these as well, although long-term data are still lacking, according to Poole at Whitehorse General.

Just 9 months after his radiator froze on Dempster Highway, Bezner returned to the Arctic. But he has added another item to his packing list: he always carries extra antifreeze for the car.

## Ute Eberle is a freelance contributor to

Chemical & Engineering News, *the independent news outlet of the American Chemical Society*.

